# Assessing blood culture appropriateness in solid organ transplant recipients: a diagnostic stewardship approach

**DOI:** 10.1017/ice.2025.10312

**Published:** 2025-12

**Authors:** Julie M. Steinbrink, Nitin Mehdiratta, Heather Pena, Ian Welsby, Amanda Seidenfeld, Adam DeVore, Matthew Ellis, Lindsay King, John Reynolds, Matthew Hartwig, Jacob Schroder, Debra Sudan, Barbara Alexander, Manuela Carugati, Jessica Seidelman

**Affiliations:** 1 Division of Infectious Diseases, Department of Medicine, Duke University School of Medicine, Durham, NC, USA; 2 Division of Critical Care Medicine, Department of Anesthesiology, Duke University School of Medicine, Durham, NC, USA; 3 Heart Services, Duke University Medical Center, Durham, NC, USA; 4 Division of Cardiothoracic Anesthesiology, Duke University Medical Center, Durham, NC, USA; 5 Division of Cardiology, Department of Medicine, Duke University School of Medicine, Durham, NC, USA; 6 Division of Nephrology, Department of Medicine, Duke University School of Medicine, Durham, NC, USA; 7 Division of Gastroenterology, Department of Medicine, Duke University School of Medicine, Durham, NC, USA; 8 Division of Pulmonary, Allergy and Critical Care, Department of Medicine, Duke University School of Medicine, Durham, NC, USA; 9 Division of Cardiovascular and Thoracic Surgery, Duke University School of Medicine, Durham, NC, USA; 10 Division of Abdominal Transplant Surgery, Duke University School of Medicine, Durham, NC, USA

## Abstract

**Objective:**

Diagnostic stewardship of blood culture utilization is important to mitigate the risks associated with unnecessary culturing. Although blood culture algorithms have been studied previously, there is a lack of data on their application among solid organ transplant (SOT) recipients. This study aims to retrospectively apply a blood culture algorithm (initially developed for a non-immunocompromised population) to adult SOT recipients and assess its performance.

**Methods:**

We conducted a manual retrospective review of adult SOT recipients with a blood culture event (BCE) between February 2022 and January 2024 at a single academic medical center. BCEs were categorized as appropriate, inappropriate, or lacking documentation, according to a previously established institutional blood culture algorithm.

**Results:**

Of 737 BCEs among adult SOT recipients, 185 (25%) were inappropriate. Within the subset of inappropriate BCEs, 178 (96%) yielded negative cultures, while 7 (4%) were deemed contaminants. No true positives were identified. Inappropriate BCEs were most commonly triggered by isolated fever and/or leukocytosis (136, 74%), and lower urinary tract infection (17, 9%). 17 of 18 BCEs due to donor blood culture positivity at the time of organ transplantation resulted in a negative blood culture in the recipient.

**Discussion:**

Once applied retrospectively, our institutional blood culture algorithm did not miss any true positive bloodstream infections among adult SOT recipients. This study provides initial evidence supporting the cautious application of blood culture diagnostic algorithms in adult SOT populations. Further prospective investigations are warranted to validate these findings.

## Introduction

Blood cultures provide critical diagnostic data on the etiology of invasive infections, leading to swift and appropriate antimicrobial therapy. However, unnecessary blood cultures obtained in clinical scenarios associated with low risk for bloodstream infections can yield false-positive results and increase antibiotic days of therapy, diagnostic interventions, and length of hospital stay.^1,2^ Thus, stewardship of such testing is imperative.^3^ Blood culture stewardship is defined as the appropriate use of blood cultures to improve patient outcomes and resource utilization. When used correctly, blood culture stewardship can mitigate the risks associated with unnecessary culturing.^4^ There are multiple safety goals for this stewardship initiative, including reducing unnecessary collection of blood cultures, avoiding contamination events, and preventing potential harms such as inappropriate antimicrobial administrations. This work is also resource-saving—decreasing the need for blood culture bottles and microbiology efforts, as well as phlebotomy costs. Blood culture stewardship proved to be especially important during the recent BD BACTEC blood culture media bottle shortage.^5^

Blood culture stewardship efforts have proven to be successful in the literature in immunocompetent hosts. Prior work by our center and others have looked at blood culture utilization in multiple high priority hospital units, including emergency department and intensive care units.^6–10^ With these interventions, blood culture utilization could be reduced by up to 30–40% without an increase in adverse effects, including patient mortality rates, hospital length of stay, and patient readmissions, along with ongoing compliance with Centers for Medicare and Medicaid Services Severe Sepsis and Septic Shock Early Management Bundle (SEP-1) core measures.^2,6,11,12^

However, there is a paucity of data regarding blood culture diagnostic stewardship interventions among immunocompromised patients, with only two published interventional studies in the literature.^13,14^ The Robinson et al. study did not involve solid organ transplant (SOT) patients, but did focus on adult hematology-oncology patients with febrile neutropenia. The Fabre et al. study included SOT patients, but did not describe how many SOT patients were included or outcomes specific to this patient population. In addition, several retrospective studies among hematology-oncology patients^15^ and stem cell transplant recipients^16–22^ questioned the utility of surveillance blood cultures among asymptomatic patients. That said, delayed antimicrobial coverage in the setting of bloodstream infections is associated with increased morbidity and mortality, especially among immunocompromised hosts, and this must be taken into consideration during stewardship efforts.^23^ To the best of our knowledge, no blood culture diagnostic stewardship manuscripts dedicated solely to the SOT population have been published. While SOT recipients are at increased risk for infection due to their immunocompromised status and the disruption of anatomical barriers occurring at time of transplant surgery, historically, blood cultures are often ordered for clinical scenarios that do not carry a high risk of bacteremia and thus are unlikely to change clinical management.^2,24,25^ In this study, we retrospectively applied a diagnostic stewardship-focused blood culture algorithm among SOT recipients and we evaluated its performance in this immunosuppressed cohort.

## Methods

### Study design

This was a retrospective study of adult SOT recipients seen in the emergency department or inpatient hospital units from February 2022 to January 2024 at a single academic medical center (Duke University Hospital, Durham, North Carolina), and for whom at least one blood culture event (BCE) (see *Definitions*) was documented during their hospital encounter. A previously published blood culture diagnostic algorithm was adapted from the Fabre et al. study at our institution and retrospectively applied to the patients included in the study without additional adaptations (**Supplemental** Figure 1). This study was approved by the University Health System’s Institutional Review Board.

### Definitions

A BCE was defined as the collection of at least one blood culture set for a specific clinical indication on the same calendar day. Based on the blood culture diagnostic algorithm mentioned above, patient charts were reviewed to categorize BCEs as appropriate (if the algorithm agreed with culture), inappropriate (if collected outside the algorithm), or lacking sufficient documentation. Additionally, if a clinical indication was not included on the original algorithm (eg, neutropenic fever), then the BCE was categorized as appropriate. Culture results were adjudicated retrospectively by a multidisciplinary panel of infectious diseases, anesthesia, emergency medicine, cardiology, and surgical physicians and advanced practice providers. Prior to adjudication, reviewers each attended an hour long synchronous virtual teaching session that reviewed the background for the project, the protocol for reviewing patient charts, entering data, and allowed time for additional questions. Reviewers were not blinded to the results of the blood cultures. Two providers reviewed each BCE. If there was disagreement or question of clinical indication or appropriateness, a third reviewer was asked to adjudicate the case. We defined thoracic organs as heart and lung, and abdominal organs as kidney, liver, pancreas, or small bowel. Blood culture results were defined as a “True Negative” if no growth was recorded in any of the blood sites; “True Positive” if growth was recorded in at least one bottle from at least one culture set taken and treated with antimicrobials; and “Contaminant” if growth was recorded in at least one bottle from the blood culture sets taken, but not felt to be clinically significant by the treating medical team and not treated with antimicrobials. This definition does differ from the Center for Disease Control’s (CDC) definition of “Contaminant,”^26,27^ as this definition was based not solely on the growth of a commensal organism on only one set of blood cultures, but was also based on the decision to treat that blood culture by the medical team.

### Data collection

BCEs at Duke University Hospital were identified using an existing surveillance blood culture database with patient-level data. Additional variables, including blood culture indication, antimicrobial treatment, maximum temperature on the calendar day of BCE, maximum white blood cell count on the calendar day of BCE, and transplant date were extracted by study team members from electronic medical records. Of note, in cases of patients receiving multiple SOTs during the study period, the most recent transplant date prior to the BCE was recorded. Lastly, we also calculated days from transplant to BCE to ascertain if a particular inflammatory reaction was closely related to a transplant surgery.

### Outcomes

The primary outcome was to describe the proportion of “true positive,” “true negative,” and “contaminant” among the high-, medium-, and low-risk clinical scenarios in a blood culture algorithm. Secondary outcomes were to stratify the primary outcome among the categories of SOT.

### Statistical analysis

Data was reported as percentages, medians, and interquartile ranges (IQR). Statistics were performed through Microsoft Excel and GraphPad Prism.

## Results

### Demographics

Four-hundred forty-seven adult SOT recipients were included in the study (Table 1). Of these, 191 were female (43%) and 256 were male (57%), with a median age of 59 years (IQR 49-66 years). The types of SOTs included 147 (33%) hearts, 99 (22%) kidneys, 95 (21%) lungs, 76 (17%) livers, 3 (1%) small bowels, 1 (<1%) pancreas, and 26 (6%) multiple organs.

**Table 1.** Characteristics of adult solid organ transplant (SOT) recipients with at least one blood culture event (BCE)

Demographic	N (%) or Median (IQR)
Males	256 (57%)
Age	59 yr (49–66 yr)
Maximum white blood cell count	13.4 × 10^3^/μL (8.1–21.4 × 10³/μL)
Maximum temperature	98.8° Fahrenheit (98.2–99.8° Fahrenheit)
BCEs adjudicated as appropriate	552 (75%)
True negative BCEs	647 (88%)
True positive BCEs	62 (8%)
Contaminant BCEs	28 (4%)
Time from transplant to BCE	225 d (24–1,846 d)

### Blood culture events

**Figure 1.**
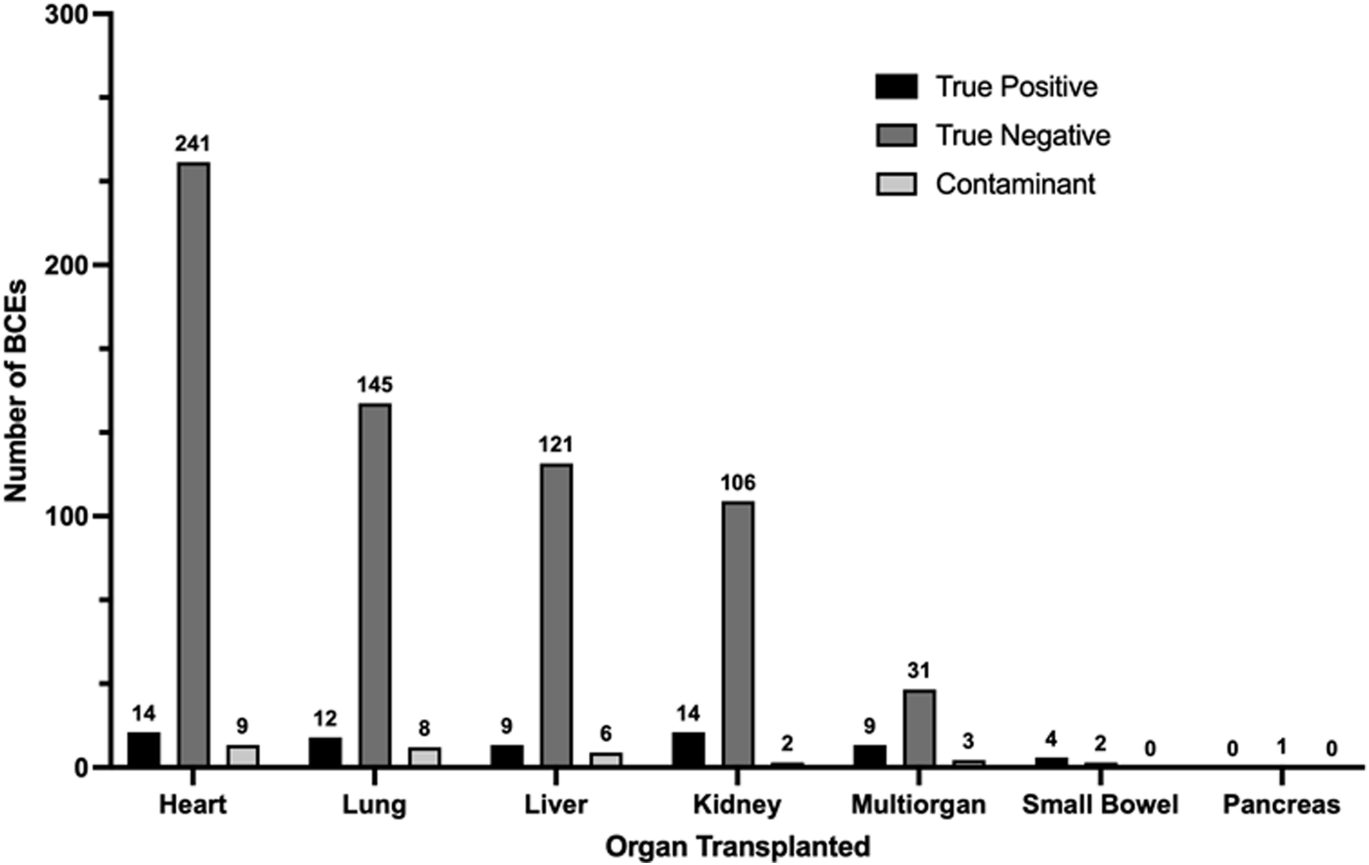
The number of blood culture events (BCEs) among adult solid organ transplant recipients stratified by organ group, as well as breakdown by status of appropriateness.

Among the 447 adult SOT recipients included in the study, 737 total BCEs were recorded during the study period. 239 BCEs (37.9%) occurred in the ED and the remaining 458 BCEs (62.1%) occurred on an inpatient unit. BCEs were documented in heart (264, 35.8%), lung (165, 22.4%), liver (136, 18.5%), kidney (122, 16.6%), multiorgan (43, 5.8%), small bowel (6, 0.8%), and pancreas (1, 0.1%) organ recipients (Fig. 1). The range of BCEs from time of transplant was 0 to 11,657 days, with a median of 225 days (IQR 24-1,846 days). A single BCE was documented in the study period for 309 (69.1%) SOT recipients, while multiple BCEs were documented in the remaining 138 (30.9%) SOT recipients (for a total of 439 BCEs occurring more than once per subject). The average BCEs for the cohort was 1.6. Of the multiple BCEs, clearance of bacteremia was a frequent indication for blood culture collection (46/439, 10.5%).

### Appropriateness of blood culture events

Of all BCEs, 552 (75%) were appropriate and 185 (25%) inappropriate. Inappropriate BCEs were generally more common among liver transplant recipients than among other organ transplant recipients (liver 35% compared to kidney 25%, lung 23%, and heart 23%) (Fig. 1). Inappropriate BCEs were frequently triggered by isolated fever and/or leukocytosis (136, 74%) in all organ groups. Of the fever and/or leukocytosis cohort of inappropriate liver BCEs, 54% were in the setting of elevated liver function tests (though 53% of those were overall downtrending liver function tests). Lower urinary tract infections were another common indication for inappropriate BCEs (17, 9%) in abdominal organ transplant recipients (Table 2). Other indications for inappropriate BCEs included pneumonia outside of protocol indications, documentation of bloodstream clearance of protocol indications, non-severe skin and soft tissue infection, and preoperative screening.

**Table 2.** Clinical indications triggering inappropriate blood culture events (BCEs) among adult solid organ transplant (SOT) recipients, stratified by organ group

	Organ transplanted					
	Heart	Liver	Lung	Kidney	Multi organ	Pancreas
Isolated fever/leukocytosis (N, %)^*^	43 (70.5)	35 (72.9)	34 (89.5)	17 (54.8)	6 (100)	1 (100)
Non-severe pneumonia (N, %)	6 (9.8)	5 (10.4)	0 (0)	1 (3.2)	0 (0)	0 (0)
Non-severe SSTI^**^ (N, %)	3 (4.9)	1 (2.1)	1 (2.6)	0 (0)	0 (0)	0 (0)
Postoperative fever (N, %)	1 (1.6)	1 (2.1)	1 (2.6)	0 (0)	0 (0)	0 (0)
Severe pneumonia (N, %)	1 (1.6)	0 (0)	0 (0)	0 (0)	0 (0)	0 (0)
Documenting clearance of bacteremia (N, %)	0 (0)	1 (2.1)	2 (5.3)	0 (0)	0 (0)	0 (0)
Lower urinary tract infection (N, %)	0 (0)	4 (8.3)	0 (0)	13 (41.9)	0 (0)	0 (0)
Preoperative screening (N, %)	0 (0)	1 (2.1)	0 (0)	0 (0)	0 (0)	0 (0)
Unclear documentation (N, %)	7 (11.5)	0 (0)	0 (0)	0 (0)	0 (0)	0 (0)

* All percentages are by organ transplanted.

** Within 48 hours from surgery.

Note. SSTI, skin and soft tissue infection.

### True positive, true negative, and contaminant blood culture events

**Figure 2.**
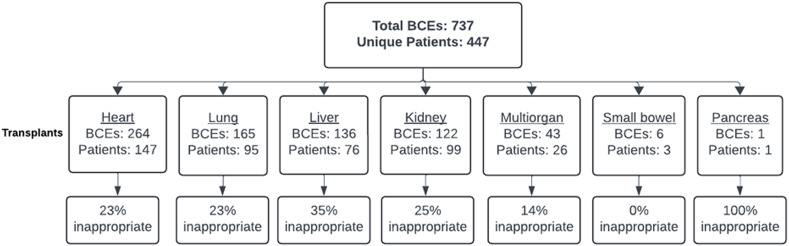
True positive, true negative, and contaminant blood culture events (BCEs) among adult solid organ transplant recipients, stratified by organ group.

Review of all (both appropriate and inappropriate) BCEs during the study period revealed 647 (88%) true negative cultures, 62 (8%) true positive cultures, and 28 (4%) contaminants. Within the subset of inappropriate BCEs (n = 185), 178 (96%) were true negatives, while 7 (4%) were contaminants. All true positive BCEs were identified as appropriate by the blood culture algorithm. For each organ transplant population, the total number of true negative BCEs significantly exceeded the number of true positive and contaminant BCEs (Fig. 2).

Of the 62 true positive BCEs, 20 (32%) occurred during the index transplant hospitalization at a median of 30 days posttransplant (IQR 7–50 days). Index hospitalization true positive BCEs occurred in 8 lung transplant recipients, 5 heart transplant recipients, 2 liver transplant recipients, and 5 multiorgan transplant recipients. Of the 42 true positive BCEs that did not occur during the index hospitalization, median time from transplant to BCE was 1,458 days (IQR 230–5,049 days).

The prevalence of true positive BCEs (excluding those collected solely to document blood culture clearance) approximately followed previously described proportions for different clinical risk scenarios, with 52% positivity in high-risk scenarios, 36% positivity in medium-risk scenarios, and 12% positivity in low-risk scenarios.^2^ As part of these determinations, “donor positive blood cultures” was included in the high-risk scenario and “neutropenic fever” was included in the medium-risk scenario.

### Blood culture events and donor-derived infections

Nineteen (2.6%) BCEs in SOT recipients resulted from concerns for potential donor-derived bloodborne pathogen transmission. Specifically, 18 BCEs were prompted by donor blood culture positivity at the time of transplant and 1 BCE occurred due to initial concern for possible infective endocarditis in the donor but with ultimately negative donor blood cultures. Of the 18 BCEs due to donor blood culture positivity, 17 (94%) resulted in negative BCEs in the recipients. The 1 positive recipient BCE did not isolate the same organism as the donor blood culture. This was treated as a true positive. Additionally, of the 18 BCEs due to donor blood culture positivity, 10 (56%) were either not on relevant antimicrobials prior to culture collection or targeted antimicrobials were started on the day of BCE, while 8 (44%) recipients were on appropriate antimicrobial coverage at the time of BCE (either for standard prophylaxis or targeted toward the donor blood culture pathogen).

## Discussion

This study highlights the potential impact of implementing a blood culture algorithm to significantly reduce blood culture collection rates in transplant patients, without compromising the detection of true positives. Notably, it contributes important preliminary data on diagnostic stewardship in immunocompromised populations—specifically SOT recipients—where such evidence is currently limited in the published literature. To date, few studies have evaluated this approach in immunocompromised patients, focusing on adult hematology-oncology patients with febrile neutropenia. One study demonstrated a 53% reduction in total blood culture collections. Importantly, it also showed that blood culture positivity was 16% on day 1 of febrile neutropenia but dropped to just 2% on days 2–12, indicating low diagnostic yield from repeat cultures in the absence of clinical changes. These findings are supported by retrospective studies involving hematology-oncology patients^15^ and stem cell transplant recipients.^16–22^ Given the recent shortages of blood culture bottles, this type of diagnostic stewardship is especially timely and carries important implications for both patient care and healthcare resource utilization.

We found that during our study period, up to 25% of BCEs in adult SOT recipients were inappropriate according to the study blood culture algorithm. Within the subset of inappropriate BCEs, 96% yielded negative cultures, while 4% were contaminants. At the same time, importantly, the algorithm did not miss any true positive BCEs, despite its application to an immunocompromised population. Total BCEs were more common in thoracic organs (heart and lung) than abdominal organs (liver and kidney) while inappropriate BCEs were more common among liver transplant recipients than among other transplant recipients. 77.6% of inappropriate BCEs among liver transplant recipients were for isolated fever and/or leukocytosis. However, for all individual large volume organ transplant populations, the percent of inappropriate BCEs remained high, up to about 25%–35%. Inappropriate BCEs occurred due to multiple different causes, but most commonly were collected in the setting of isolated fever and/or leukocytosis. In this setting for inappropriate liver BCEs in particular, about 40% occurred after recent biliary intervention and about 50% were in the setting of elevated liver function tests (both of which may have raised concern for possible bacteremia by the clinical teams).

The range of BCEs from time of transplant was wide, with a median of 225 days, with only 32% of true positive BCEs occurring during the index hospitalization, despite their higher immunocompromised status with transplant induction therapy and the disruption of anatomical barriers occurring at time of transplant surgery. These earlier true positive BCEs were more commonly seen in thoracic and multiorgan transplant recipients than in abdominal organ recipients. The proportion of true positive and true negative BCEs during the index hospitalization were similar at about 33%, though the overall number of true negative BCEs was much larger.

These data also suggest reconsidering the practice of routinely drawing blood cultures in SOT recipients during the index transplant hospitalization solely due to positive donor blood cultures, though a larger sample size will be needed for further analysis. Literature suggests that up to 5% of organ donors may be bacteremic at the time of procurement.^28–31^ Untreated transmission can result in severe complications including graft loss, prolonged hospitalization, and increased mortality. However, if bacteremia is known pretransplant, donors frequently receive a period of appropriate antimicrobial coverage prior to procurement, with a follow-up targeted antimicrobial treatment course in the recipient. In this setting, bacterial transmission can be prevented without deleterious effect to the organ recipient or allograft, regardless of recipient blood culture collection posttransplant.^29,30^ Of the 18 BCEs in our study due to donor blood culture positivity, 94% were negative and did not change clinical management, and 100% of recipient BCEs did not result in the same pathogen growth as donor cultures. Additionally, these culture results did not appear to be affected by peri-transplant antimicrobials. Of the 18 recipient BCEs, 56% were either not on appropriate antimicrobials prior to culture collection or antimicrobials were started on the day of BCE, and thus likely did not influence culture clearance.

This study does have some limitations, including its single center, retrospective approach. Also, we were unable to obtain data on the volume of blood collected in each blood culture bottle to understand how many true positives may have been missed due to low-volume blood culture collection. Moreover, reviewers were not blinded to the results of the blood cultures, which may have biased their adjudication of appropriateness. Additionally, our definition of contaminant differed from the CDC’s standard definition in an attempt to identify as many possible true positive blood cultures that would be missed by utilizing the algorithm. Another limitation is the lack of clinical data in this population supporting the categorization of certain clinical scenarios (e.g., neutropenic fever) into appropriate/inappropriate based on positivity rates. Furthermore, the study was not designed to examine and compare safety signals and outcome measures within the recipients, such as hospital length of stay or mortality. Lastly, in the setting of donor blood culture positivity, only recipient BCEs were reviewed, this study did not investigate the possibility of other potentially donor-derived invasive infection complications in organ recipients outside of bacteremia.

In conclusion, this study highlighted that a diagnostic stewardship-focused blood culture algorithm did not miss any true positive bacteremias in SOT recipients and provided initial evidence supporting the cautious application of the algorithm in this population, where data is severely needed. Further investigation is warranted to validate these findings and optimize diagnostic stewardship strategies for improved patient outcomes.

## Supporting information

Steinbrink et al. supplementary material Steinbrink et al. supplementary material

## Supplementary material

To view supplementary material for this article, please visit https://doi.org/10.1017/ice.2025.10312
