# Supplementary figures and images for: Assessing blood culture appropriateness in solid organ transplant recipients: a diagnostic stewardship approach
Source: Infect Control Hosp Epidemiol. 2025 Sep 29;46(12):1222–7. doi: 10.1017/ice.2025.10312 (PMC12483178; doi:10.1017/ice.2025.10312)

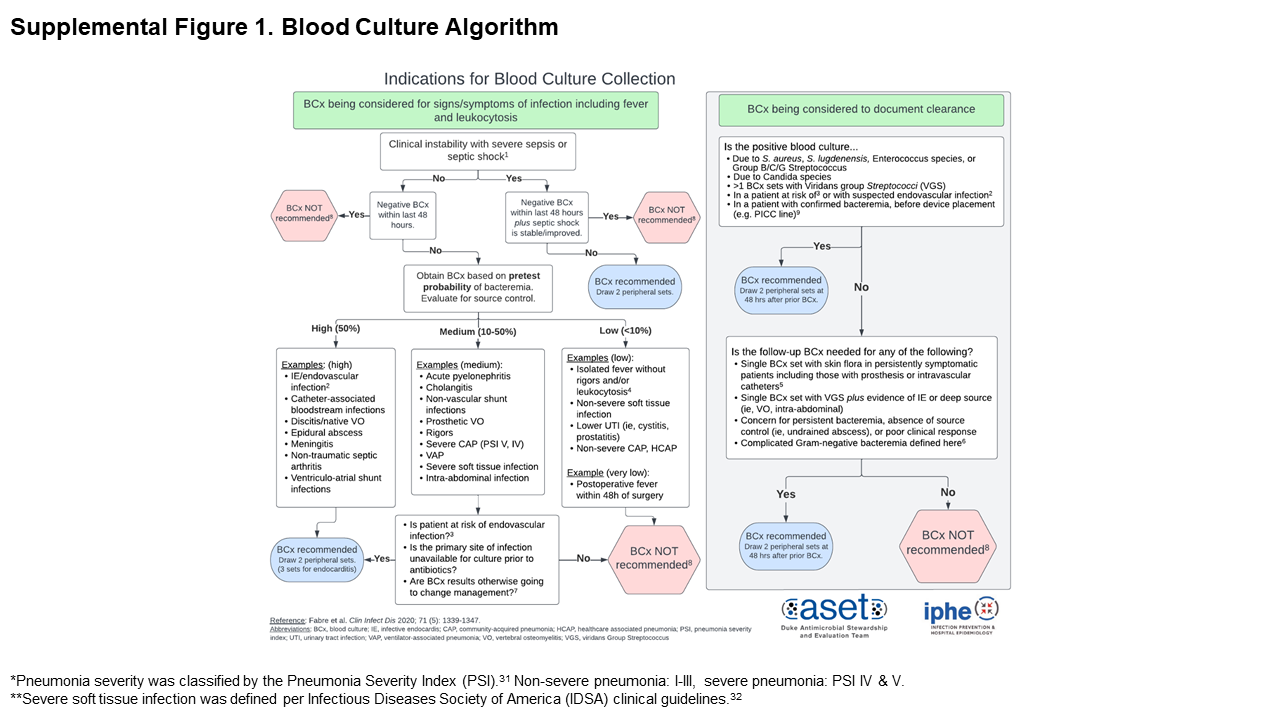

Supplement: Steinbrink et al. supplementary material [file S0899823X25103127sup001.tiff]
